# Validation of the multi-day Boston remote assessment of neurocognitive health (BRANCH) among cognitively impaired & unimpaired older adults

**DOI:** 10.1016/j.tjpad.2025.100057

**Published:** 2025-01-13

**Authors:** Emma L. Weizenbaum, Stephanie Hsieh, Cassidy Molinare, Daniel Soberanes, Caitlyn Christiano, Andrea M․Román Viera, Juliana A.U. Anzai, Stephanie Moreno, Emily C. Campbell, Hyun-Sik Yang, Gad A. Marshall, Reisa A. Sperling, Kathryn V. Papp, Rebecca E. Amariglio

**Affiliations:** a Department of Neurology, Brigham and Women's Hospital, Boston, MA, 02115, USA; b Department of Neurology, Massachusetts General Hospital, Harvard Medical School, Boston, MA, 02129, USA

**Keywords:** Digital cognitive assessment, Mild cognitive impairment, Preclinical Alzheimer's disease

## Abstract

**Background:**

The multi-day Boston Remote Assessment of Neurocognitive Health (BRANCH) is a remote, web-based assessment designed to capture the earliest cognitive changes in the preclinical stage of Alzheimer's disease (AD). It has been validated in unimpaired older adults, but as individuals progress on the AD continuum, assessments need to remain feasible and valid at different clinical stages. The focus of this study was to assess feasibility and validity of multi-day BRANCH in participants with and without cognitive impairment.

**Methods:**

For seven days participants completed the BRANCH paradigm to capture a muti-day learning curve score. Participants also completed the mini-mental-status-exam (MMSE) and the Quick Dementia Rating Scale (QDRS). The primary cohort included 81 older adults: 38 with cognitive impairment (CI) and 43 cognitively-unimpaired (CU). A complementary replication cohort included 16 participants with consensus-defined mild cognitive impairment (MCI) and 47 demographically-matched cognitively unimpaired participants.

**Results:**

Multi-day BRANCH was feasibile with 92 % or participants completing all seven days of testing. More CI than CU reported nervousness and found tasks slightly less enjoyable on Day 1, but ratings increased at a similar rate in both groups. Convergent validity was confirmed by a positive association between BRANCH and total MMSE and QDRS scores. There was a large effect size of group status on BRANCH (CI vs. CU; *Cohen's* d = 0.83) and per logistic regression, BRANCH significantly predicted group status (β = -1.49, *p* < 0.001); even more so between MCI and CU in the replication cohort.

**Conclusions:**

Findings suggest that a remotely administered web-based assessment of multi-day learning is feasible and valid in participants with and without cognitive impairment.

## 1 Introduction

There is a need for cognitive measures to be both feasible and sensitive to a dynamic range of performances across the early Alzheimer's disease (AD) spectrum to support early detection and longitudinal tracking, particularly in the context of ongoing AD secondary prevention trials [1]. Evidence suggests that specific memory processes, such as paired associative learning, may be particularly sensitive to differences in cognition at the earliest stages of AD [2,3], and can be measured through learning of repeated exposure (LORE) to the same material [4,5,6]. Multiple studies have shown that individuals with mild cognitive impairment (MCI) show decreased LORE compared to cognitively unimpaired individuals [7,8]. Additionally, this pattern has been observed in cognitively unimpaired older adults with elevated amyloid who had reduced LORE when tested over the course of several months, compared to those with non-elevated amyloid [4].

Building off of this literature, we have developed and previously validated the Boston Remote Assessment of Neurocognitive Health (BRANCH), a remote, unsupervised mobile assessment of associative memory, designed to capture cognitive processes affected in preclinical AD that are otherwise challenging to detect using traditional measures [9]. As smartphone use in older adults has expanded exponentially in the past few decades [10], digital remote assessment on computers and mobile devices is an increasingly viable method of cognitive assessment that warrants acceptability and feasibility testing in individuals with varying degrees of cognitive function. Until now, our work has only evaluated this form of testing in cognitively unimpaired older adults, and feasibility of the current paradigm remains unknown in those with cognitive impairments. Assessing individuals with daily testing of repeated stimuli over one week using the BRANCH platform allows for quantification of personalized multi-day learning curves. Previously, we have demonstrated, in cognitively unimpaired individuals, that the multi-day BRANCH paradigm is feasible with high levels of adherence and acceptability, is reliable and stable when repeated one month later, and is valid with strong convergence with traditional in-person cognitive testing [11]. Further, diminished multi-day learning curves collected over one week were reduced in cognitively unimpaired individuals with elevated amyloid compared to non-elevated amyloid [12]. However, for the assessment of learning curves to be useful for the duration of a secondary prevention trial, in which participants might progress to MCI, the BRANCH paradigm must also be feasible among those who are cognitively impaired.

The current study seeks to expand upon our previous validation work by exploring the psychometrics of multi-day BRANCH in those with cognitive impairments. Specifically, we sought to assess feasibility and validity of the multi-day BRANCH paradigm in individuals with cognitive impairment (CI) compared to demographically-matched cognitively unimpaired (CU) participants from a cross-sectional community-based study cohort. While feasibility of remote digital assessments in those with CI has been previously demonstrated [13], we were particularly interested in the feasibility and overall performance of multi-day BRANCH in a CI group. We hypothesized that the multi-day BRANCH assessment would be feasible for those with CI to complete remotely, and that any differences in adherence and acceptability between CU and CI participants would not impact the validity of the measure. We also hypothesized that multi-day BRANCH scores would reflect convergent validity with traditional clinical measures and construct validity with the expectation that multi-day BRANCH performance would be able to differentiate and predict group cognitive status (CU vs. CI). Finally, we sought to test replicability of these validity hypotheses in a smaller but highly-characterized sample of participants with a clinical-consensus diagnosis of MCI or cognitive unimpairment.

## 2 Methods

### 2.1 Participants from the primary community-based cohort

The primary sample was recruited from the ongoing community-based Memory & Aging Cohort (MAC) Study with a limited amount of background or baseline data. MAC participants were initially recruited from the community and BWH cognitive neurology clinic, with a broad inclusion criteria that includes people characterized as CU, MCI, or mild dementia from any causes. The MAC study protocol was approved by the Mass General Brigham (MGB) Institutional Review Board (IRB), and all participants signed a written informed consent. Participants were included in the present study if they were 60 years of age or older and English-speaking.

All MAC participants completed the Mini-Mental State Examination [14] in person. All participants had a study partner (e.g., spouse, friend) who completed the Quick Dementia Rating System (QDRS). The QDRS is a 10-item questionnaire that measures cognitive and daily functioning, as well as behavior, and has been shown to yield comparable results to the Clinical Dementia Rating (CDR) in both clinical and registry sample settings [15]. In the present study, participants were classified as cognitively unimpaired (CU) if they had a QDRS score between 0 and 1, and as cognitively impaired (CI) if they had a QDRS score between 2 and 9. Of note, the QDRS scale formally classifies total scores between 2 and 5 as MCI, and score between 6 and 12 as mild dementia. Furthermore, a sensitivity analysis was run with the CI participants defined as having an MMSE total score between 24 and 27 to test whether the findings held with an alternative way of categorizing the cognitively impaired group. The list of eligible participants was filtered by those with and without cognitive impairment (using the completed QDRS scores noted above), and participants were contacted sequentially and invited to complete multi-day BRANCH until roughly 20 male and female participants had completed BRANCH in the respective CU and CI groups. Participants were asked to use their own web-enabled electronic device to log on to BRANCH, which could be either a personal computer or mobile device (smartphone or tablet). Study issued devices and WIFI-connectivity were available if needed, but all participants used personal devices in the current study. During initial and daily instructions, participants were asked to complete the BRANCH tests on the same device throughout the study days.

### 2.2 Participants from the complementary replication cohort

The MAC study-determined clinical diagnosis, desribed above, was solely based on the informant-reported QDRS without clinical consensus review. Thus, to validate our findings from the MAC study, we included a complementary replication cohort made up of several additional longitudinal studies in which participants’ study diagnosis was designated through clinical consensus meetings, specifically: the Harvard Aging Brain Study (HABS) (2P01AG036694–11; R.A.S., K.A.J.), Instrumental Activities of Daily Living Study (IADL) (R01AG053184; G.A.M.), and the Massachusetts Alzheimer's Disease Research Center (MADRC) Longitudinal Cohort Study (P30 AG062421; B.T.H.). All of the research MCI and CU participants received a full neuropsychological battery and clinical assessments (e.g., CDR) as part of the ongoing observational studies that were used in determining the research diagnosis.

### 2.3 Multi-day boston remote assessment of neurocognitive health (BRANCH)

The BRANCH platform and multi-day paradigm used in the present study have previously been described in detail [9], [11] and will be summarized here. All participants were given the multi-day version of the BRANCH battery that is comprised of three different tests: the Face-Name Test, the Groceries-Prices Test, and the Digit-Signs Test, all of which involve the same stimulus pairs repeated over seven days. In the Face-Name Test, participants are shown 20 face-name pairs and later asked to recall the first letter of the names of the faces and to match a given face to its correct name. In the Groceries-Prices Test participants were shown pairs of items and prices and later asked to match a given grocery item with its correct price. Lastly, on the Digit-Signs Test participants indicate (yes-no) whether a presented street sign matches one of the six digit-sign pairings shown at the top of the screen; the objective is to complete as many pairing determinations as possible in 90 seconds. As included in our previous work using multi-day BRANCH [11], at the end of each test administration participants are asked to complete several brief survey questions on the enjoyability of the three subtests (0 – not enjoyable to 10 – very enjoyable), the type of device used (i.e., smartphone, tablet, or computer), and whether contextual factors were present such as environmental distractions, technical difficulties, pain, nervousness, and/or fatigue. Participants completed the assessment once a day for seven days in response to daily email or text notifications sent at a participant-designated time. Participants were included in the final analysis if they completed at least 4 out of the 7 study days, which was previously shown to be the number of days needed to see differences in performance between amyloid positive and amyloid negative cognitively unimpaired participants [12]. The complete multi-day BRANCH course of participation was required to occur within a 14-day window.

All participants underwent informed consent. Study procedures were conducted in accordance with human subjects’ protections and the study protocol was approved by the Mass General Brigham Institutional Review Board.

### 2.4 Statistical analyses

The primary outcome from the multi-day BRANCH paradigm is a learning curve composite of the three subtests. To best capture the acquisition of information over seven days we have used a multi-day learning curve (MDLC) metric derived from an adjusted area-under the curve score for each participant based on their performance across the study period, previously described in detail [11]. Descriptive statistics, T-tests, and Pearson correlations were used to evaluate the feasibility of this paradigm as it relates to participant characteristics in the given sample, as well as the correlations between multi-day learning curves and the MMSE and total QDRS scores. We also examined construct validity of BRANCH by assessing differences in the effect size of group status (CU vs. CI) on MDLC performance, and similarly the use of the MDLC as a predictor of group status in a logistic regression. All statistical analyses were completed using R (v4.0.3). Data from this study is accessible in the Harvard Aging Brain Study public dataset v2.0: https://habs.mgh.harvard.edu/researchers/data-details/

## 3 Results

### 3.1 Participant characteristics from the primary community-based cohort

We included 81 participants from the primary cohort (MAC) who met the criteria described above (mean age:70 ± 7 years old; 51 % female). Baseline characteristics are displayed in Table 1. Sex, age, and education were matched during recruitment and did not significantly differ between CU and CI groups*.*

**Table 1:** Multi-day BRANCH Participant Characteristics by Group.

	Primary Cohort		Replication Cohort	
Characteristic	**CU**, *n* = 43^1^	**CI**, *n* = 38^1^	**CU**, *n* = 47^1^	**MCI**, *n* = 16^1^
Age	70 (8) (60,101)	70 (5) (60,83)	75 (7) (61,92)	76 (9) (62,95)
Sex				
Female	21 (49 %)	20 (53 %)	24 (51 %)	8 (50 %)
Male	22 (51 %)	18 (47 %)	23 (49 %)	8 (50 %)
Ethnicity				
Hispanic or Latino	0 (0 %)	1 (2.6 %)	0 (0 %)	0 (0 %)
Race				
Asian	0 (0 %)	3 (7.9 %)	2 (4.3 %)	1 (6.2 %)
Black or African American	0 (0 %)	3 (7.9 %)	4 (8.5 %)	1 (6.2 %)
Native Hawaiian or Other Pacific Islander	0 (0 %)	0 (0 %)	0 (0 %)	1 (6.2 %)
White	43 (98 %)	31 (82 %)	41 (87 %)	13 (81 %)
Years of Education	17.1 (1.26) (16,20)	17.11 (1.78) (12,20)	15.17 (1.91) (12,18)	15.25 (1.91) (12,18)
MMSE Score	29 (1) (25,30)	28 (3) (16,30)	29.11 (0.8) (27,30)	26.50 (2.61) (20,30)
Total QDRS Score	0.14 (0.35) (0.00, 1.5)	3.39 (1.37) (2.00, 8.00)	NA	NA
CDR Global Score	NA	NA	0.04 (0.14) [0, 0.5]	0.69 (0.51) [0.5, 2.5]
Device				
Computer	22 (51 %)	12 (33 %)	30 (64 %)	9 (56 %)
Smartphone/Tablet	21 (59 %)	26 (68 %)	17 (36 %)	7 (44 %)

Note. Abbreviations: CU = Cognitively Unimpaired; CI = Cognitively Impaired; MCI = Mild Cognitive Impairment; MMSE = Mini-Mental Status Examination; CDR = Clinical Dementia Rating; QDRS = Quick Dementia Rating System.

1 Mean (SD) (Range); n(%).

### 3.2 Feasibility

Participants were invited to take BRANCH on any device with internet access, but were asked to keep the device used consistent throughout the study period. The CU group was evenly split between their use of a mobile-device (17 smartphones, 4 tablets) (49 %) vs. PC (51 %). In comparison, the CI group tended to use mobile devices (23 smartphones, 3 tablets) (68 %), which reflected a trend, this preference did not surpass significance by group (χ^2^ = 2.42, df=1, *p* = 0.112).

A total of four participants were not included in current analyses due failure to complete the seven study days within the requisite 14-day window. In the CU group, one participant completed only five days of testing, while the remainder of CU participants completed all seven days. In the CI group, two participants completed five or six days, respectively, while the remainder completed all seven days of testing. The mean number of days to complete the learning curve was 7.14 days (sd=0.52) in the CU group and 7.58 days (sd=1.7) in the CI group which reflects a non-significant trend towards longer study completion lengths in CI participants due to somewhat less consecutive study-day completion (*t* = −1.53, df = 42.99, p-value = 0.13). The average minutes to complete the daily assessment collapsed across study days was marginally, but statistically, longer in the CI group (13.37+/- 1.82 min) compared to the CU group (12.49+/- 1.63 min) (*t* = −2.01, df = 54.35, p-value = 0.01).

The presence of technical difficulties were reported as occurring equally by both CU participants (14.4 % of assessments) and CI participants (14.3 % of assessments), (*t* = −0.423, df = 119.23, p-value = 0.67). The most frequently endorsed technical difficulty was poor device response to finger-taps (11 % in both groups; *t* = −0.49, df = 119.88, p-value = 0.62). All other reported technical difficulties were endorsed in less than 3 % of assessments for both groups.

**Fig. 1:**
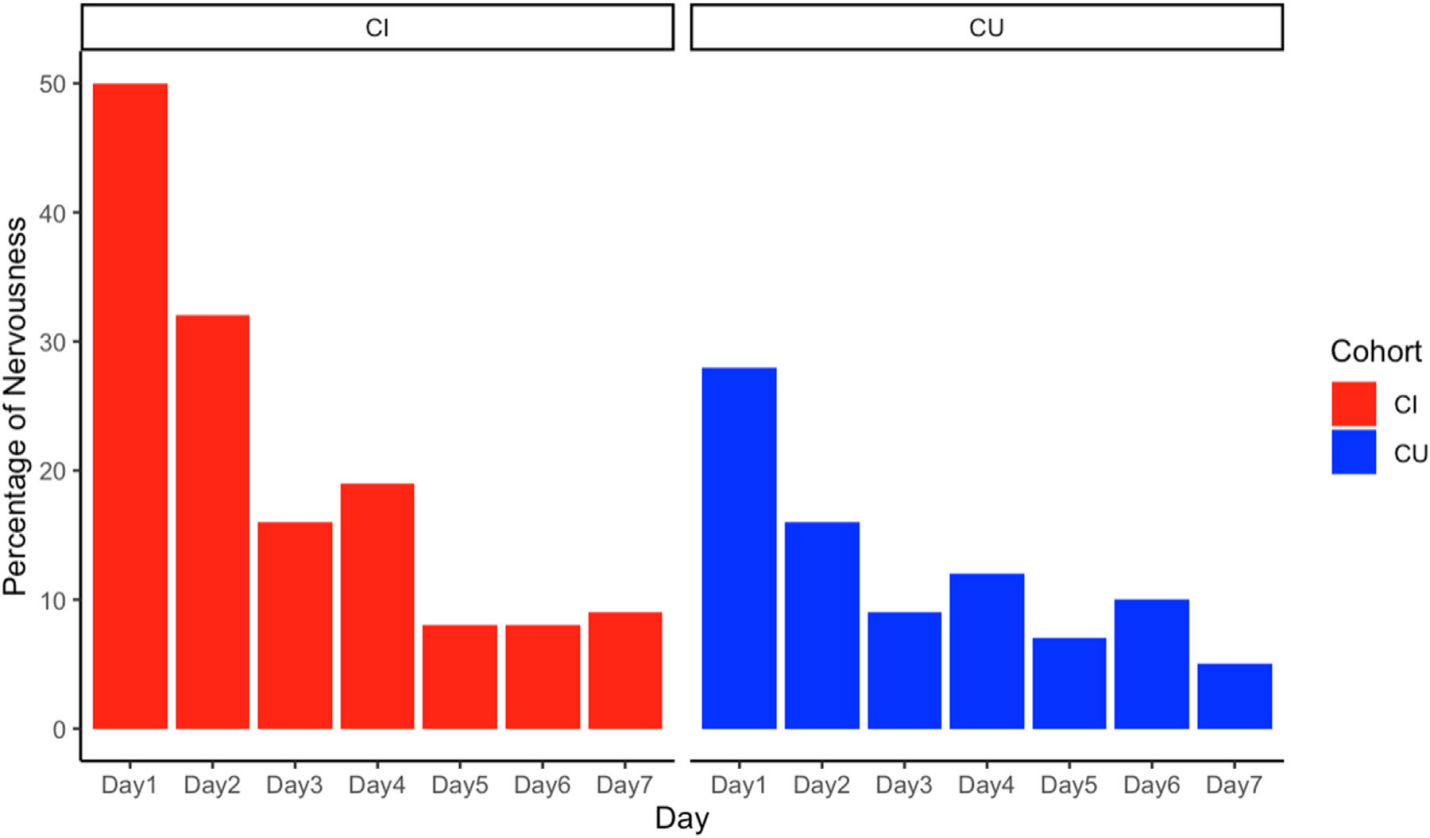
Percentage of Participants Endorsing Nervousness by Day by Group in a Community-Based Sample Note. Participants were asked to endorse if they felt nervous at the time of their testing – a higher percentage of CI participants endorsed nervousness significantly more frequently on Days 1 and 2 but endorsement decreased to comparable frequencies over course of the seven days. Abbreviations: CU = Cognitively Unimpaired; CI = Cognitively Impaired.

The most frequently reported physical symptom or emotion present during a given assessment across the seven days was nervousness. CI participants endorsed nervousness as present during 20 % of their assessments compared to only 12 % of the time by CU participants (*t*= −0.24, df= 137.35, p-value=0.81). As shown in Fig. 1, the greatest differences between groups in the frequency of reported nervousness were on Days 1 and 2, but both groups showed a decrease in nervousness over the seven study days. Fatigue was endorsed slightly, but not significantly more often in CI participants (8.5 % of assessments) compared to CU participants (7.7 % of assessments) (*t* = 1.45, df= 128.72, p-value=0.15), and pain was only endorsed during 1 % or fewer of the assessments in either group. A general “other physical or emotional factors present during testing” checkbox was selected by CU participants during 7 % of assessments and during 9 % of assessments completed by CI participants (*t* = 0.75, df= 133.17, p-value=0.45).

**Fig. 2:**
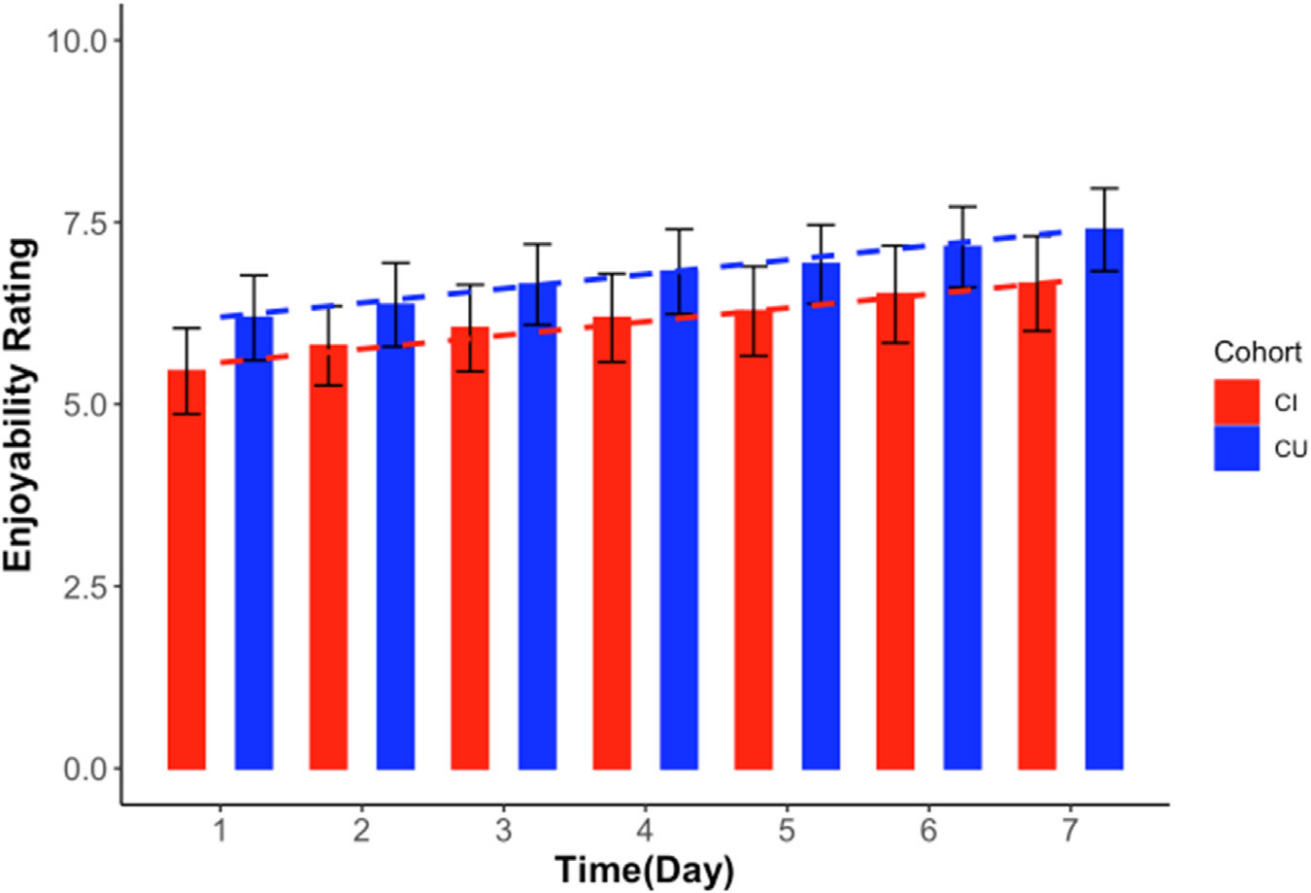
Self-Reported Task Enjoyability Ratings Across the Seven Study Days in a Community-Based Sample Note. While CI participants initially reported the multi-day BRANCH as less enjoyable than the CU participants on Day 1 (*p* < 0.001), both groups’ rated the assessments as increasingly enjoyable over the seven days. Abbreviations: CU = Cognitively Unimpaired; CI = Cognitively Impaired.

Additionally, participants rated how much they had enjoyed the tasks at the end of each daily assessment using a 1–10 point likert scale (1 being ‘Not Enjoyable’,10 being ‘Very Enjoyable’). Overall, participant enjoyability increased over the seven days in both groups (Fig. 2). However, more specifically, CI participants found the assessment less enjoyable on the first day of testing compared to CU participants (β= 0.2, *p*=<0.001), but no group differences was seen in their rate of increased enjoyability across the study period (β=−0.04, *p* = 0.175). Given the range of MMSE scores seen in participants with a QDRS score of 2–9 (mild impairment), a sensitivity analysis was run on CI participants within this sample with an MMSE of 24–27 (*n* = 18), and the above findings of feasibility held in regard to both study completion and acceptability in this smaller sample.

### 3.3 Convergent and construct validity

**Fig. 3:**
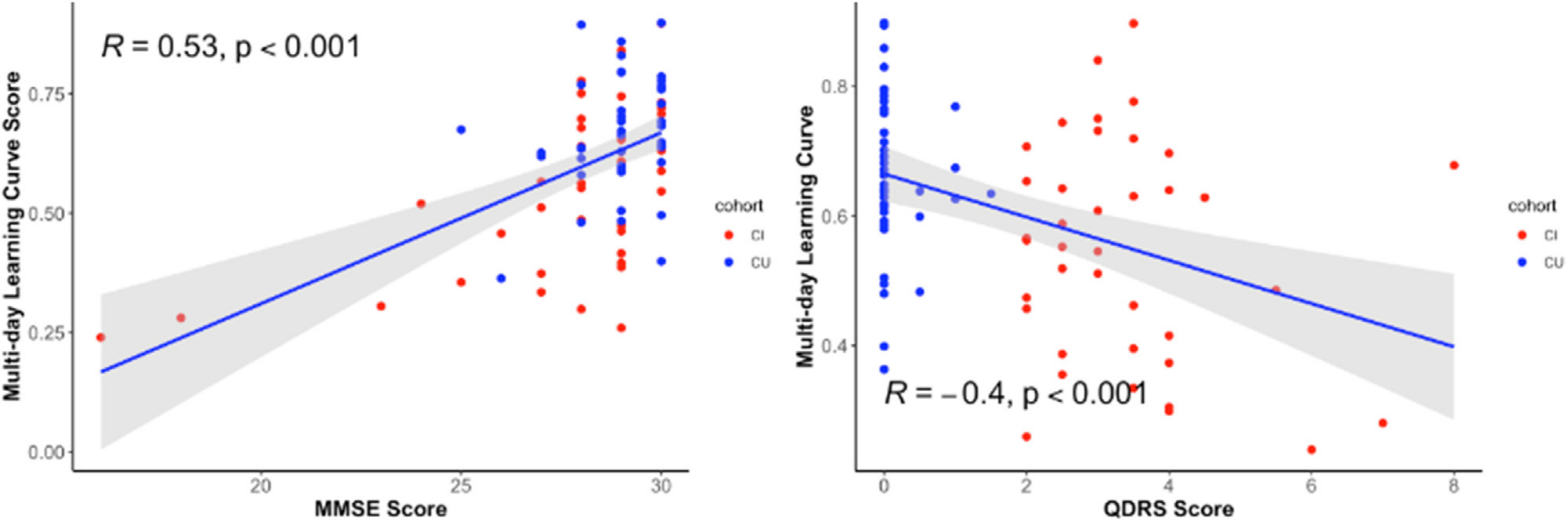
Association Between Multi-day BRANCH Score and Traditional Cognitive Screeners in a Community-Based Sample Note. The multi-day BRANCH learning curve score was significantly correlated with traditional cognitive screening measures (lower score on MMSE and higher score on QDRS indicated greater impairment). Abbreviations: CU = Cognitively Unimpaired; CI = Cognitively Impaired; MMSE = Mini-Mental Status Examination; QDRS = Quick Dementia Rating System.

As a means towards evaluating convergent validity with existing traditional measures of cognition and function, we examined the correlation between the MDLC composite score (comprising an average of the three BRANCH subtests) and the MMSE and QDRS total scores. We found that across all participants, better BRANCH performance (i.e., higher MDLC composite score) was significantly associated with a higher MMSE total score (*r* = 0.53, *p* < 0.001) and a lower QDRS total score (with higher scores reflecting worse cognitive functioning) (*r* = −0.40, *p* < 0.001) (Fig. 3). The lower QDRS score association was largely driven by CI participants given the greater range that defined the characterization of this group (QDRS of ≥2).

**Fig. 4:**
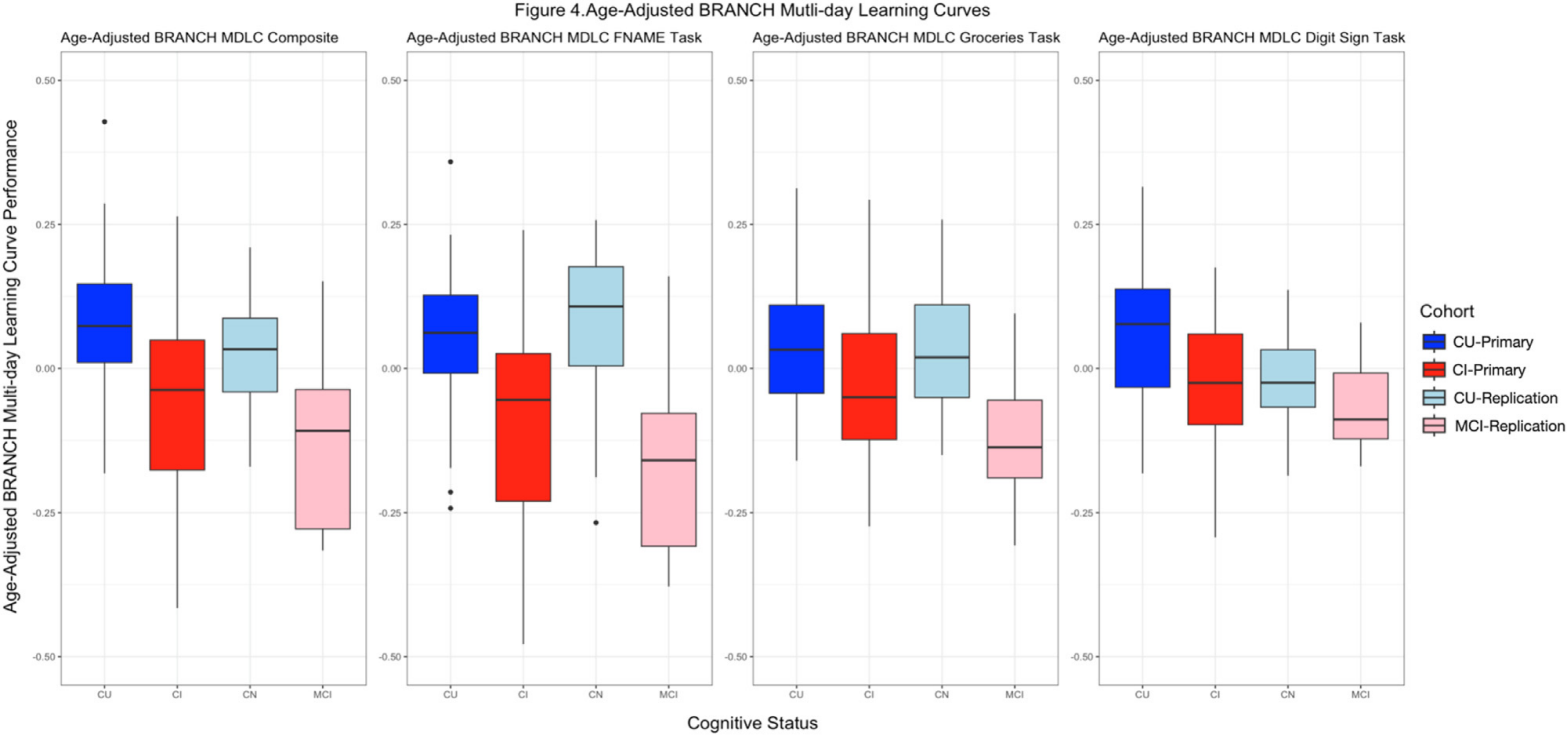
Differentiation on the BRANCH Multi-Day Learning Curve Performance for Each Task by Group (Primary and Replication Cohorts) Note. The 7-day multi-day learning curve scores are shown for each participant group in the primary and replication cohorts for the three individual tasks of the multi-day BRANCH assessment, as well as the composite score. The learning curve scores shown here were calculated with age as a covariate given that the replication sample was on average five years older. Abbreviations: MDLC = multi-day learning curve CU = Cognitively Unimpaired; CI = Cognitively Impaired; MCI = Mild Cognitive Impairment.

The construct validity of BRANCH was assessed by measuring differences in performance on the measure between CU and CI participants. CU participants had significantly higher mean composite MDLC scores compared to CI participants with a large effect size (mean score =0.67/1.0, sd = 0.13 vs. 0.54/1.0, sd = 0.16; *Cohen's* d = 0.83) (Fig. 4). Additionally, it was found that the MDLC composite significantly predicted participant group status (i.e. CI vs. CU) using logistic regression (β = −1.49, CI (−2.21, −0.78), *OR* = 0.23, *p* < 0.001); . The same differentiation was found when comparing groups on the individual measures that comprise the MDLC composite including Face-Name (β = −1.25, CI (−1.82, −0.69), *OR* = 0.29, *p* < 0.001), Groceries-Prices (β = −0.72, CI (−1.47, 0.02), *OR* = 0.49, *p* = 0.05), and Digit-Signs (β = −1.34, CI (−2.08, −0.60), *OR* = 0.26, *p* = 0.001). The sensitivity analysis of CI participants with a restricted MMSE range of 24–27 (*n* = 18) also found significant group differentiation between this restricted group and CU participants on the MDLC composite and three subtasks.

### 3.4 Replication cohort from complementary observational sample

As previously noted, we were interested in knowing whether the above feasibility and validity results from a minimal-contact community-based sample could be replicated in a highly-characterized observational study sample (HABS/IADL/ MADRC Longitudinal Cohort Study). Using a 3:1 ratio using the ‘MatchIt’ package in R, a group of 47 cognitively unimpaired (CU) participants (derived from a total sample of 180 CU) were selected to match sample characteristics (sex, age, and education) of the 16 MCI participants from this replication cohort who had previously completed BRANCH. This resulted in a combined replication study sample of 63 participants (*n* = 47 CU and *n* = 16 MCI), age:75 ± 8 years old; 51 % female; 15.2 ± 1.9 years of education). Baseline characteristics are displayed in Table 1. Overall, this sample was similar in sex distribution, but roughly five years older (mean age of 75 vs. 70) and slightly less educated (mean of 15 vs. 17 years of education). In the replication cohort, MCI participants had on average a one point lower total MMSE score than the CI participants in the primary sample (CI =27.51 (sd=3); MCI=26.5 (sd=2.6), p-value = 0.049). There were no differences between the mean MMSE score between CU participants in the replication vs. primary cohorts – both groups had a mean MMSE of 29.0.

Similar to the primary cohort, the replication cohort participants were invited to take BRANCH on any device with internet access, but to remain with a consistent device throughout the study period. In the replication CU group, 43 % of participants chose to use a mobile device vs. a PC, and a similar split was seen in MCI participants with 44 % using mobile devices vs. PCs. In the replication CU group, one participant completed only five days of testing, while the remaining 46 CU participants completed all seven days. In the MCI group, all 16 of the participants completed the full seven days of testing. The mean number of days to complete the learning curve was 7.8 days (sd=1.78) in the CU group and 8.0 days (sd=1.79) in the MCI group, which was not a statistically significant difference (*t* = −0.45, df = 27.02, p-value = 0.65). The average minutes to complete the daily assessment collapsed across study days was marginally, but statistically, longer in the MCI group (13.46+/- 1.42 min) compared to the replication CU group (12.80+/- 1.78 min) (*t* = −1.32, df = 18.13, p-value = 0.02).

The presence of technical difficulties was reported with a similar frequency in both replication groups, but slightly lower in MCI: CU participants (12.4 % of assessments) and MCI participants (10.9 % of assessments). The most frequently endorsed technical difficulty was poor device response to finger-taps (CU = 8 %; MCI = 7 %). Other reported technical difficulties were endorsed as present in less than 5 % of assessments for both groups. Both CU and MCI participants endorsed nervousness as present during 9 % of their assessments, and fatigue was also endorsed at the same frequency in both groups at 8 %. Pain was endorsed during 1 % of assessments by the MCI group and not endorsed by any participants in the CU group. A general “other physical or emotional factors present during testing” checkbox was selected by CU participants during 8 % of assessments and during 18 % of assessments completed by MCI participants (*t*= −5.07, df= 42.04, p-value<0.001).

Additionally, participants in the replication cohort, rated how much they had enjoyed the FNAME tasks at the end of each daily assessment using a 1–10 point likert scale; enjoyability ratings were not collected for this cohort for the other two tasks. Overall, participants from both groups equally enjoyed the FNAME task (Average FNAME rating enjoyability: CU=5.67 (sd=2.36); MCI=5.34 (sd=2.33)) and there were no group differences in the rate of increased enjoyability across the study period (*t* = 1.25; df=178.29; p-value=0.21).

Towards replicating the previously seen convergent validity in the primary cohort, lower total MMSE scores were associated with lower BRANCH MDLC composite scores in this replication cohort (MCI and CU combined) (*r* = 0.58, *p* < 0.001).We also examined the construct validity of multi-day BRANCH by comparing the MDLC scores between the CU and MCI participants in this replication cohort. Similar to the results seen in the primary cohort, MDLC composite scores of the CU participants were significantly higher than the MCI participants with a large effect size (mean score =0.57/1.0, sd = 0.10 vs. 0.41/1.0, sd = 0.16; *Cohen's* d = 1.30). As shown in Fig. 4, the pattern of higher performance scores in CU participants and lower scores in CI participants is reflected in both the primary and replication cohorts. The replication cohort's mean age is five years older than the primary cohort, and our previous work has shown age effects with older participants performing worse on BRANCH measures [11]. As such, the MDLC scores in this figure are calculated adjusting for age so that a comparable pattern between cohorts could be depicted on the same axis in this figure.

Additionally, the MDLC composite significantly predicted participant group status (i.e. CU vs. MCI) using logistic regression (β = −2.33 CI (−3.16, −1.51), *OR* = 0.10, *p* < 0.001). The same differentiation was found when comparing groups on the individual measures that comprise the MDLC composite including Face-Name (β= −1.53, CI (−1.99, −1.08), *OR* = 0.22, *p* < 0.001), Groceries-Prices (β = −1.79, CI (−2.43, −1.14), *OR* = 0.17, *p* < 0.001), and Digit-Signs (β = −1.64, CI (−2.91, −0.36), *OR* = 0.19 p = 0.013).

## 4 Discussion

The current study sought to assess the feasibility and validity of the multi-day BRANCH paradigm in multiple cohorts of participants with and without CI. In concordance with our prior work using BRANCH, examining only CU individuals [9], [11], [12], the present study demonstrated feasibility with high rates of adherence and study completion as well as acceptability of the assessment method in CI participants in both the primary and replication cohorts. Interestingly, contrary to the concerns often articulated related to mobile device use in older adults [16], our study found that in the primary sample, there were higher rates of smartphone vs. computer use in CI participants compared to CU participants. In the smaller replication sample, both MCI (*n* = 16) and CU (*n* = 47) tended to choose to use a PC vs. mobile device at a similar rate, just over 50 %.

When completing the assessments, we asked participants to indicate the presence of other contextual factors. In our primary cohort, CI participants reported more frequently feeling anxious at the time of the assessment compared to CU, especially in the first two days of the study. Although follow up questions about the nature or source of the anxiety were not asked, it could be related to self-awareness of being assessed in an area of relative weakness, namely memory. This finding also aligns with the existing literature indicating higher rates of anxiety in older adults with cognitive impairment [17], [18], [19]. This finding may also be driven by some QDRS items relating to mood and behavior changes in addition to cognitive changes, and thus leading to the CI group having greater base rates of anxiety. In the replication sample, although skewed by numbers of participants per group, MCI participants endorsed anxiety at similar rates to CU.

We also assessed the acceptability of completing a remote assessment for seven days, and asked participants to rate how enjoyable they found the tests after each day's assessment. Although CI participants in the primary sample initially endorsed lower enjoyability ratings on Day 1, on average, ratings by both CI and CU participants increased at a similar rate over the seven days. Compared to other ecological momentary assessment (EMA) burst-style remote assessments, the structure of multi-day BRANCH is unique in that participants gain increased familiarity with the exact same stimuli over the course of a week. As such, participants may perceive the improvement in their performance, which could be contributing to the increased sense of enjoyability. In the MCI vs. CU replication, there were no differences in enjoyability ratings between groups. This finding underscores the acceptability of multi-day BRANCH as a repeated remote assessment that can be used and tolerated by participants with varying levels of cognitive function. Moving forward, it could be valuable to know whether providing participants with explicit indications of their performance increases adherence rates and enjoyability ratings of BRANCH.

As hypothesized, poorer learning curve performance on multi-day BRANCH was associated with more severe cognitive symptom ratings on the QDRS. Additionally, in both our primary and replication cohorts, MMSE scores were significantly correlated with learning curve performance across participants with and without cognitive impairment. The present findings are concordant with our past work demonstrating an association, in cognitively unimpaired older adults, between learning curves and scores on the in-lab Preclinical Alzheimer's Cognitive Composite-5 (PACC-5) and the self and care-partner reported Cognitive Functioning Index (CFI) [11]. The present findings both replicate and expand upon this prior study by demonstrating the convergent validity of the multi-day BRANCH in CI participants as well.

In order to ensure validity of the multi-day BRANCH paradigm we sought to see whether there were expected group differences in performance between CU and CI participants recruited from a community-based sample. There was a large effect size when comparing the differences in performance between these groups’ using the composite multi-day learning curve scores in our primary cohort, and this finding was even more pronounced in the replication cohort with well-charactarized cognitive unimpaired and MCI participants. Similarly, a logistic regression model showed that the composite learning curve and sub-test learning curve scores all significantly predicted group status (CU vs. CI/MCI) in the primary and replication cohorts, as hypothesized.

Although the number of MCI participants in the replication cohort was significantly smaller than the number of CI in the primary cohort (16 vs. 38), the similar and even more robust pattern of group differentiation is reassuring towards establishing the convergent and construct validity of multi-day BRANCH in participants with some degree of cognitive impairment. Moving forward, we hope to address the limitations of our current sample size by studying multi-day BRANCH performance in a larger number of participants, including a sample comprised of participants with a broader spectrum of cognitive function and with increased ethnic and racial diversity so as to ensure generalizability and relevance of findings. In the future, it will be helpful to have greater characterization of symptoms and clinician-ratings of cognitive function in addition to study partner ratings. Similarly, beyond BRANCH, the only other objective cognitive data collected was the MMSE, and as such, in the future it will be useful to learn how the multi-day BRANCH paradigm relates to other cognitive measures, such as the PACC-5 in older adults with cognitive impairment. Future directions may also include identifying the nature of relationship between multi-day BRANCH learning curves and AD biomarkers such as amyloid and tau in participants longitudinally beyond the cognitive unimpaired stage [12].

The present study represents an essential stepping-stone towards identifying additional ways to feasibly and sensitively measure AD-specific cognitive processes (e.g. associative learning over repeated exposures) in asymptomatic (preclinical) to early symptomatic (MCI) individuals. In conclusion, the multi-day BRANCH's remote digital assessment was found to be feasible, acceptable, and valid in participants with and without cognitive impairment. It is hoped that this further validation of BRANCH will support the field in measuring cognitive changes at the earliest stages of AD serving the development and implementation of timely intervention.

## 5 Potential conflicts of interest

Each of the authors of this study have nothing to report relevant to the findings of this manuscript.

K Papp has served as a consultant for Biogen Idec and Digital Cognition Technologies. R.A. Sperling has received research funding from NIH, Alzheimer's Association and Eli Lilly for this research. She has served as a consultant for AC Immune, Biogen, Eisai, Janssen, Neurocentria and Roche. Her spouse has served as a consultant to Biogen, Janssen, and Novartis. G.A. Marshall has received consulting fees from Ono Pharma USA Inc. and has received research salary support for serving as site principal investigator for clinical trials funded by Eisai Inc. and Eli Lilly and Company.

## CRediT authorship contribution statement

**Emma L. Weizenbaum:** Writing – review & editing, Writing – original draft, Supervision, Project administration, Methodology, Formal analysis, Conceptualization. **Stephanie Hsieh:** Writing – original draft, Visualization, Formal analysis, Data curation. **Cassidy Molinare:** Project administration, Data curation. **Daniel Soberanes:** Project administration, Formal analysis, Conceptualization. **Caitlyn Christiano:** Investigation, Data curation. **Andrea  M․Román Viera:** Funding acquisition, Data curation. **Juliana A.U. Anzai:** Investigation, Data curation. **Stephanie Moreno:** Investigation, Data curation. **Emily C. Campbell:** Investigation, Conceptualization. **Hyun-Sik Yang:** Writing – review & editing, Funding acquisition, Conceptualization. **Gad A. Marshall:** Writing – review & editing, Funding acquisition. **Reisa A. Sperling:** Writing – review & editing, Funding acquisition, Conceptualization. **Kathryn V. Papp:** Writing – review & editing, Supervision, Methodology, Investigation, Conceptualization. **Rebecca E. Amariglio:** Writing – review & editing, Supervision, Resources, Project administration, Investigation, Funding acquisition, Conceptualization.

## Declaration of competing interest

The authors declare the following financial interests/personal relationships which may be considered as potential competing interests:

Rebecca Amariglio reports financial support was provided by National Institute of Health. Gad Marshall reports financial support was provided by National Institute of Health. Reisa Sperling reports financial support was provided by National Institute of Health. Kathryn Papp reports a relationship with Biogen Idec and Digital Cognition Technologies that includes: consulting or advisory. Reisa Sperling reports a relationship with Eli Lilly and Company that includes: funding grants. Reisa Sperling reports a relationship with AC Immune, Biogen, Eisai, Janssen, Neurocentria and Roche that includes: consulting or advisory. Gad Marshall reports a relationship with Eisai Inc. and Eli Lilly and Company that includes: employment. Gad Marshall reports a relationship with Ono Pharma USA Inc that includes: consulting or advisory. If there are other authors, they declare that they have no known competing financial interests or personal relationships that could have appeared to influence the work reported in this paper.
